# Segmental sandwich osteotomy and tunnel technique for three-dimensional reconstruction of the jaw atrophy: a case report

**DOI:** 10.1186/s40729-017-0077-3

**Published:** 2017-05-01

**Authors:** Mario Santagata, Nicola Sgaramella, Ivo Ferrieri, Giovanni Corvo, Gianpaolo Tartaro, Salvatore D’Amato

**Affiliations:** 1 Multidisciplinary Department of Medical and Dental Specialties, Oral and Maxillofacial Surgery Unit, AOU - University of Campania “Luigi Vanvitelli”, Naples, Italy; 2 Piazza Fuori Sant′Anna 17, 81031 Aversa, Italy

**Keywords:** Sandwich osteotomy, Ridge augmentation, Tunnel technique

## Abstract

**Background:**

A three-dimensionally favourable mandibular bone crest is desirable to be able to successfully implant placement to meet the aesthetic and functional criteria in the implant-prosthetic rehabilitation. Several surgical procedures have been advocated for bone augmentation of the atrophic mandible, and the sandwich osteotomy is one of these techniques. The aim of the present case report was to assess the suitability of segmental mandibular sandwich osteotomy combined with a tunnel technique of soft tissue. Based on our knowledge, nobody described before the sandwich osteotomy with tunnel technique to improve the healing of the wound and meet the dimensional requirements of preimplant bone augmentation in cases of a severely atrophic mandible.

**Case presentation:**

A 59-year-old woman with a severely atrophied right mandible was treated with the sandwich osteotomy technique filled with autologous bone graft harvested by a cortical bone collector from the ramus. Clinical examination revealed that the mandible was edentulous bilaterally from the first molar to the second molar region. Radiographically, atrophy of the mandibular alveolar ridge in the same teeth site was observed. We began to treat the right side. A horizontal osteotomy of the edentulous mandibular bone was then made with a piezoelectric device after tunnel technique of the soft tissue. The segmental mandibular sandwich osteotomy (SMSO) was finished by two (mesial and distal) slightly divergent vertical osteotomies. The entire bone fragment was displaced cranially, and the desirable position was obtained. The gap was filled completely with autologous bone chips harvested from the mandibular ramus through a cortical bone collector. No barrier membranes were used to protect the grafts. The vertical incisions were closing with interruptive suturing of the flaps with a resorbable material. In this way, the suture will not fall on the osteotomy line of the jaw; the result will be a better predictability of soft and hard tissue healing.

**Conclusions:**

Segmental mandibular sandwich osteotomy is an easy and safety technique that could be performed in an atrophic posterior mandible. Future studies involving long-term follow-up are needed to evaluate the permanence of these results.

## Background

In cases of atrophic mandible, the distance to the mandibular canal and the transverse decrease in bone is an anatomic limitation for prosthetic rehabilitation with dental implants. The gold standard for treatment of this mandibular atrophy continues to be autologous bone grafting [1, 2].

A relatively modern technique for vertical bone augmentation is sandwich osteotomy. Schettler and Holtermann first described this technique, with promising results [3]. The sandwich technique has been proved to be relatively safe, with successful long-term results, in both the mandible and the maxilla [4–10]. The technique is executed by segmentalising the alveolar bone through a minimal vestibular incision (similar to osteotomies such as alveolar distraction osteogenesis), transporting the segment attached to the periosteum to the desired three-dimensional planned area and fixing it with plate and screws. Most studies have used autogenous bone graft as the filler for the created gap.

To the best of our knowledge, the present study is the first to use sandwich osteotomy to obtain both vertical and transversal bone gain flapless; in more detail, the authors used the tunnel technique of the soft tissue without a full-thickness flap to perform the described mandibular osteotomy to improve hard and soft tissue healing.

## Case report

A 59-year-old woman with a severely atrophied right mandible was treated with the sandwich osteotomy technique filled with autologous bone graft harvested by a cortical bone collector from the ramus.

**Fig. 1:**
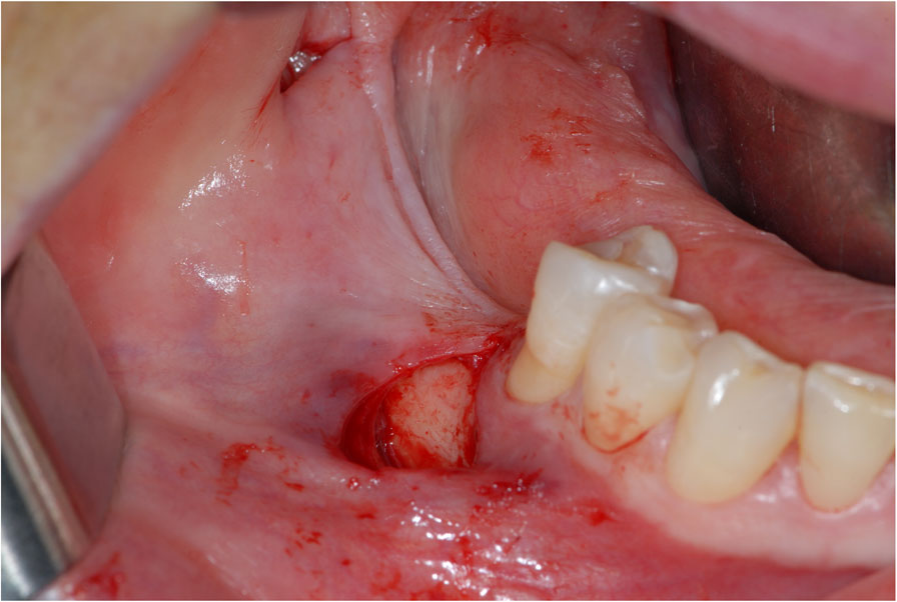
Tunnel technique

The requirements of the Helsinki Declaration were observed, and the patient gave informed consent for all surgical procedures. After local infiltration of anaesthesia (mepivacaina plus adrenaline 1:200,000), buccally and lingually to the defect area, a single vertical incision was initiated at the distal margin of the mesial tooth (43) to the defect. The second incision was carried out distally about 3 cm far from the first (Fig. 1). The soft tissues were elevated from the bone through the tunnelling mechanism cranially, mesially and distally in a subperiosteal plane. The elevator of Zucchelli (Stoma®—Storz am Mark GmbH, Emmingen-Liptingen, Germany) was used for the subperiosteal dissection.

However, the entire periosteum could be preserved to ensure adequate vascularisation of the future bone cranial segment.

**Fig. 2:**
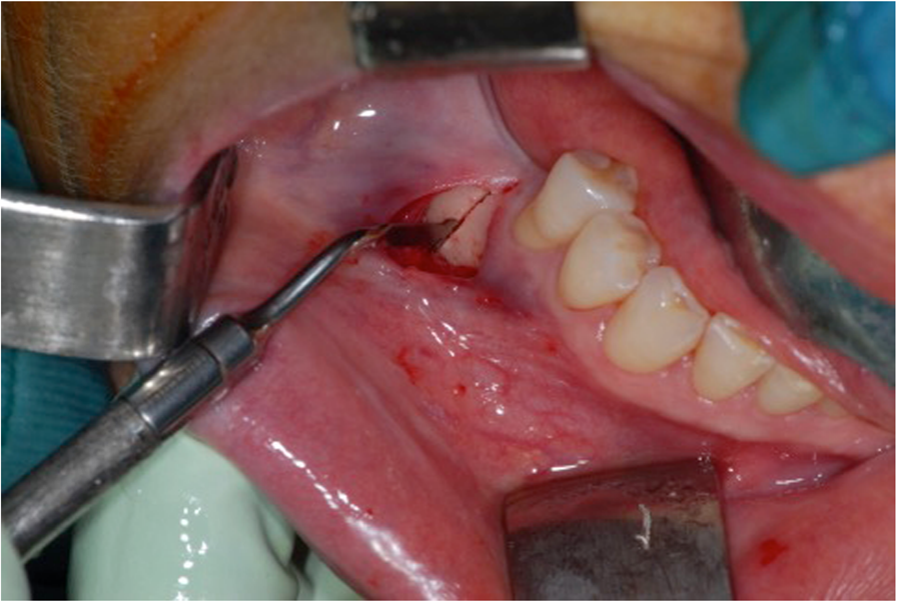
Mandibular osteotomy by piezosurgery

A horizontal osteotomy of the edentulous mandibular bone was then made with a piezoelectric device (Mectron Medical, GE, Italy). The tip MT1-10 was used to perform the osteotomy. The segmental mandibular sandwich osteotomy (SMSO) was finished by two (mesial and distal) slightly divergent vertical osteotomies (Fig. 2). The horizontal osteotomy was located at least 2 mm below the ridge bone and approximately 2 mm above the mandibular canal. The vertical mesial osteotomy was made 2 mm distal to the last tooth and 2 mm above the mental foramen. Also, the mesial vertical muco-periosteal incision is necessary to place the incision 2 mm distant from the mental foramen. The bone fragment remains anchored to the lingual and crestal periostea. The entire bone fragment was displaced cranially, and the desirable position was obtained.

**Fig. 3:**
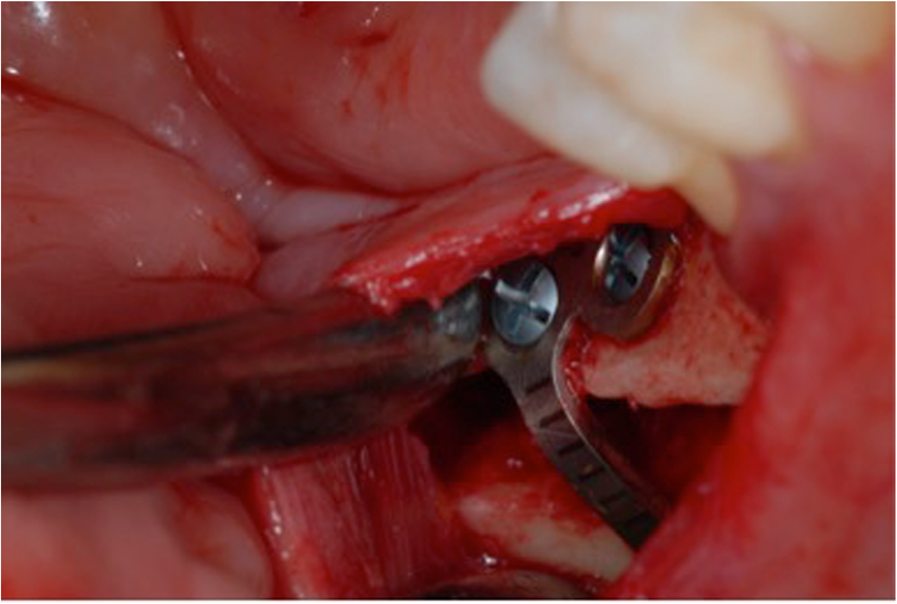
Mobilised segment moved to the desired three-dimensional position and fixed with plate and screws

**Fig. 4:**
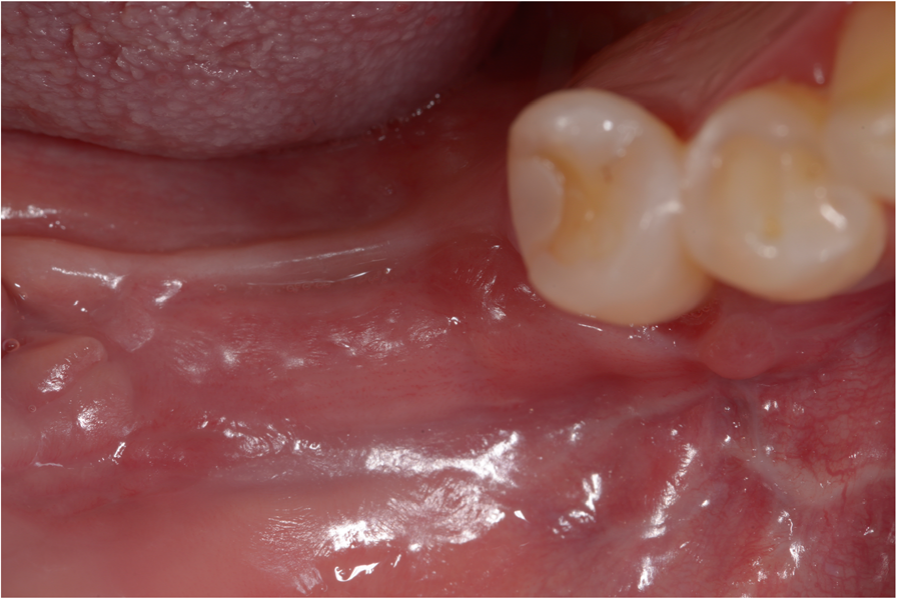
Wound healing after 2 months

**Fig. 5:**
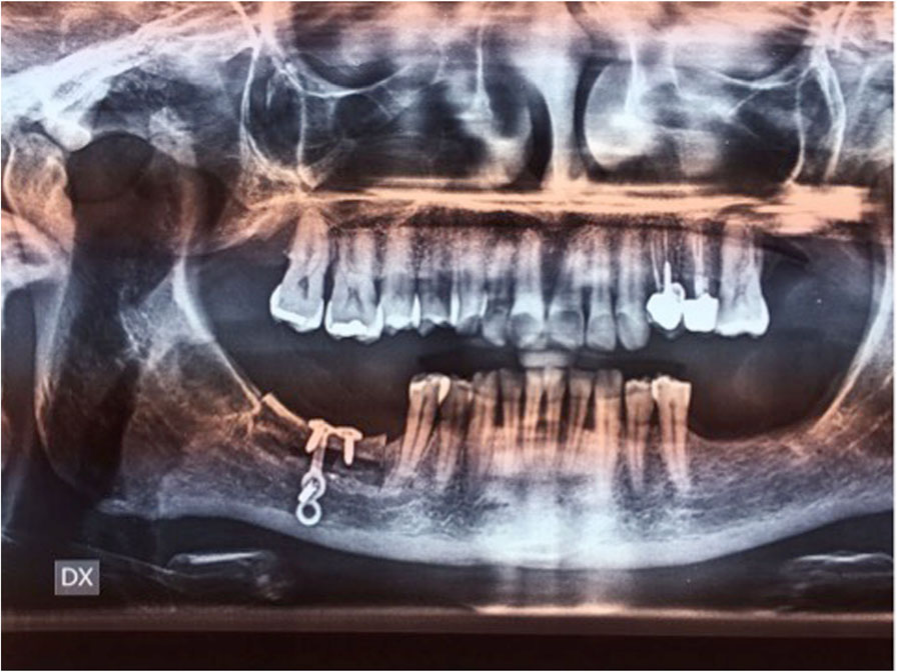
OPG postoperatively

The length of the segments was matched to the deficient, resorbed alveolar ridge. The segment was displaced crestally to the desired three-dimensional place and fixed with 0.8 mm thickness, pure titanium, L-Plate with 2.4 mm titanium matrix mandible cortex screws—self-tapping tip (Synthes GmbH Eimattstrasse, Oberdorf, Switzerland; Fig. 3). The gap was filled completely with autologous bone chips harvested from the mandibular ramus by a cortical bone collector (Safescraper Twist, Meta, Italy). No barrier membranes were used to protect the grafts. The vertical incisions were closing with interruptive suturing of the flaps with a resorbable material (Polysorb 3-0, Covidien LLC, MA, USA). In this way, the suture will not fall on the osteotomy line of the jaw; the result will be a better predictability of soft and hard tissue healing (Fig. 4). Orthopantomography (OPG) was performed immediately after the procedure (Fig. 5).

### Discussion

The sandwich technique for bone augmentation of the atrophic mandible was first described by Schettler and Holtermann, with promising results. Since then, variations in this surgical procedure have been proposed by several investigators [4–10].

All these authors have proposed the same approach about the flap: paracrestal incision. In order to preserve the blood supply, it is of fundamental importance that the least number possible of the vessels of the soft tissue be damaged. Based on this concept, we believe that changing the flap design can obtain the improvement of the healing of the wound. The sandwich osteotomy with the tunnel technique meets these requests. This is because the incisions were only two and vertical in the buccal side. Another advantage, especially with respect to implants, is that vascularisation is maintained in the bone ridge throughout augmentation intervention; thus, the interface at the implant shoulder in terms of hard-to-soft tissue to implant interface is kept as true to the original as possible.

This technique should be applied in patients with at least 5 mm of minimal crestal amount of bone above the nerve to perform the sandwich osteotomy successfully.

We prefer to obtain not more than 5 mm of the vertical movement for the sandwich graft. Efforts to displace the segment greater than 5 mm not only risk the potential for vascular embarrassment by detaching periosteal blood supply but also can excessively rotate the segment palatally, compromising aesthetic gingival projection.

We observed no signs of impaired sensibility after the sandwich osteotomy technique. Jensen found transient paraesthesia in all patients, lasting up to 6 weeks [7].

## Conclusions

In conclusion, segmental mandibular sandwich osteotomy is an easy and safety technique that could be performed in atrophic posterior mandible.

Future studies involving long-term follow-up are needed to evaluate the permanence of these results.
